# Identifying Early Responders to Dry Needling for Lower-Limb Spasticity in Multiple Sclerosis: A Secondary Responder Analysis of a Pilot Randomized Controlled Trial

**DOI:** 10.3390/brainsci16020240

**Published:** 2026-02-21

**Authors:** Alberto Javier-Ormazábal, Marta González-Sierra, Montserrat González-Platas

**Affiliations:** 1 Division of Physiotherapy, Hospital Universitario de Canarias, Carretera Ofra S/N, 38320 San Cristóbal de La Laguna, Santa Cruz de Tenerife, Spain; 2 Physiotherapy Department, Universidad Europea de Canarias, C. Inocencio García, 1, 38300 La Orotava, Santa Cruz de Tenerife, Spain; marta.gonzalez@universidadeuropea.es; 3 Department of Physical Medicine and Pharmacology, Universidad de La Laguna, C/Sta. María Soledad S/N, 38071 La Laguna, Santa Cruz de Tenerife, Spain; 4 Division of Internal Medicine, Hospital Universitario de Canarias, Carretera Ofra S/N, 38320 San Cristóbal de La Laguna, Santa Cruz de Tenerife, Spain; 5 Division of Neurology, Hospital General de Fuerteventura Virgen de la Peña, Carretera del Aeropuerto, Km 1, 35600 Puerto del Rosario, Las Palmas, Spain; montserrat.gonzalezplatas@gmail.com

**Keywords:** multiple sclerosis, spasticity, dry needling, treatment outcome, neurologic rehabilitation

## Abstract

**Highlights:**

**What are the main findings?**

- Approximately one-third of ambulatory people with multiple sclerosis showed clinically meaningful improvement after a single session of dry needling for lower-limb spasticity.
- Early responders were more likely to have a relapsing–remitting disease course and lower baseline disability.

**What are the implications of the main findings?**

- Dry needling may be a beneficial adjunctive intervention for a specific subgroup of patients with multiple sclerosis rather than a universal treatment.
- Responder-based analyses can support personalized rehabilitation strategies and inform patient selection in future clinical trials.

**Abstract:**

**Background/Objectives:** Response heterogeneity limits the implementation of dry needling for spasticity in multiple sclerosis (MS). This secondary analysis aimed to identify early responders and explore predictors of response. **Methods:** We conducted a responder analysis of a pilot randomized, double-blind, sham-controlled trial (NCT05956119) including 18 ambulatory MS patients with spasticity, randomized to a single session of dry needling (*n* = 9) or sham (*n* = 9). Sensitive responder criteria were defined as improvement ≥ 2.0 s in Timed Up-and-Go, ≥5 points in MSQOL-54 physical component, or ≥10% in 25-Foot Walk Test at 4 weeks. **Results:** Using these criteria, 33.3% (3/9) of dry needling recipients were classified as responders versus 0% (0/9) in the sham group (*p* = 0.214). Responders were more frequently observed among participants with relapsing–remitting MS (100% vs. 40%, *p* = 0.090) and lower baseline disability (Expanded Disability Status Scale 3.4 vs. 4.4). A positive association was observed between baseline pyramidal subscore and physical quality-of-life change, although this did not reach statistical significance (r = 0.52, *p* = 0.150) in the active group. **Conclusions:** Approximately one-third of participants met predefined responder criteria following dry needling; however, these findings should be interpreted as preliminary signals derived from an exploratory, underpowered pilot analysis. These results are hypothesis-generating and require confirmation in adequately powered trials.

## 1. Introduction

Multiple sclerosis (MS) is a chronic inflammatory, demyelinating, and neurodegenerative disease of the central nervous system characterized by marked clinical heterogeneity and an unpredictable disease course [1]. Variability in lesion distribution, disease phenotype, and rate of progression leads to a wide range of motor and non-motor symptoms, even among individuals with comparable demographic and clinical profiles [2]. Common manifestations include muscle weakness, fatigue, sensory disturbances, impaired balance, and spasticity, the latter affecting up to 80% of people with MS at some point during the disease course [3,4,5]. Spasticity represents a major contributor to gait dysfunction, reduced mobility, and limitations in activities of daily living, and is consistently associated with diminished health-related quality of life and participation restrictions [6]. Although pharmacological management remains a cornerstone in the treatment of spasticity in MS, its effectiveness is often incomplete and may be limited by adverse effects or suboptimal tolerability [7].

Consequently, rehabilitation interventions play a critical complementary role by targeting neuromuscular impairments, optimizing movement efficiency, and enhancing functional performance [8]. However, response to rehabilitation is highly variable in MS, reflecting the complex interaction between disease-related factors, such as phenotype and disability level, and individual characteristics, including neuromuscular reserve and capacity for neuroplastic adaptation [9]. This heterogeneity poses substantial challenges for the evaluation and implementation of therapeutic interventions in both research and clinical practice [10].

Dry needling (DN) is a minimally invasive intervention increasingly incorporated into physical therapy practice and has recently gained attention within neurorehabilitation [11]. The technique involves the insertion of solid filiform needles into specific muscle tissues with the aim of modulating altered neuromuscular activity, reducing muscle stiffness, and normalizing dysfunctional activation patterns [12]. Mechanistically, DN has been proposed to influence peripheral and central neural excitability, spinal reflex modulation, and the mechanical properties of muscle, all of which are relevant targets in neurological conditions characterized by spasticity and impaired motor control [13].

Emerging evidence in neurological populations suggests that DN may reduce hypertonia, improve motor performance, and induce favorable changes in functional outcomes [14,15,16]. In people with MS, the available literature remains limited but includes small, randomized trials, quasi-experimental studies, and a recent double-blind randomized sham-controlled pilot trial reporting potential improvements in gait-related parameters, functional mobility, and health-related quality of life following DN applied to lower-limb musculature. Nevertheless, treatment effects appear inconsistent across individuals, and clinically meaningful benefits are not uniformly observed, indicating substantial interindividual variability in responsiveness to DN [17,18,19,20,21].

Such variability is consistent with the well-documented heterogeneity of MS-related spasticity, which may arise from differing contributions of neural, muscular, and biomechanical mechanisms across patients [22]. As a result, analyses relying solely on group-level mean differences may fail to capture clinically relevant improvements experienced by specific subgroups of individuals [23]. In recent years, responder-based analytical approaches have been increasingly advocated to complement conventional statistical methods, particularly for functional mobility and walking outcomes, as they allow the identification of changes exceeding established thresholds for clinical relevance and provide information more closely aligned with individualized clinical decision-making [24].

Accordingly, the primary aim of the present study was to identify early responders to a single session of dry needling applied to lower-limb spasticity in people with MS using a responder-based framework. Secondary aims were to explore baseline clinical characteristics associated with treatment response and to examine the relationship between baseline spasticity severity and changes in functional and quality-of-life outcomes. By focusing on individual response patterns rather than average treatment effects, this exploratory analysis seeks to generate hypotheses to inform patient selection strategies and the design of future confirmatory clinical trials.

## 2. Materials and Methods

### 2.1. Study Design and Participants

This was a secondary responder analysis of a single-center, pilot, randomized, double-blind, sham-controlled trial conducted at the Neurorehabilitation Unit of the Hospital Universitario de Canarias (Spain). The original trial protocol was approved by the Institutional Ethics Committee (CHUC_2023_40) and registered prospectively at ClinicalTrials.gov (NCT05956119) on 7 July 2023. All participants provided written informed consent. The trial was conducted in accordance with the Declaration of Helsinki and reported following CONSORT guidelines [25].

The original trial was not designed or powered to detect responder differences or predictors of response. Accordingly, all analyses should be interpreted as exploratory and hypothesis-generating. Post hoc power calculations are reported solely to contextualize the observed findings.

Ambulatory patients with a definitive MS diagnosis were recruited between January 2022 and June 2023. Inclusion criteria were: age 18–65 years; diagnosis of relapsing-remitting or secondary progressive MS; Expanded Disability Status Scale (EDSS) score 2.0–6.5; presence of lower limb spasticity (pyramidal subscore ≥ 1); and ability to walk 25 feet with or without assistance. Exclusion criteria included: relapse within the previous 3 months; changes in antispastic medication in the last month; contraindications for DN (bleeding disorders, local skin infections, needle allergy); pregnancy or lactation; and participation in other interventional studies.

### 2.2. Randomization and Intervention

Participants were randomly allocated (1:1) to either real DN or sham intervention using a computer-generated random sequence with variable block sizes (4 and 6). An independent researcher not involved in assessment or treatment maintained the allocation sequence. Both participants and outcome assessors remained blinded throughout the study.

**Figure 1:**
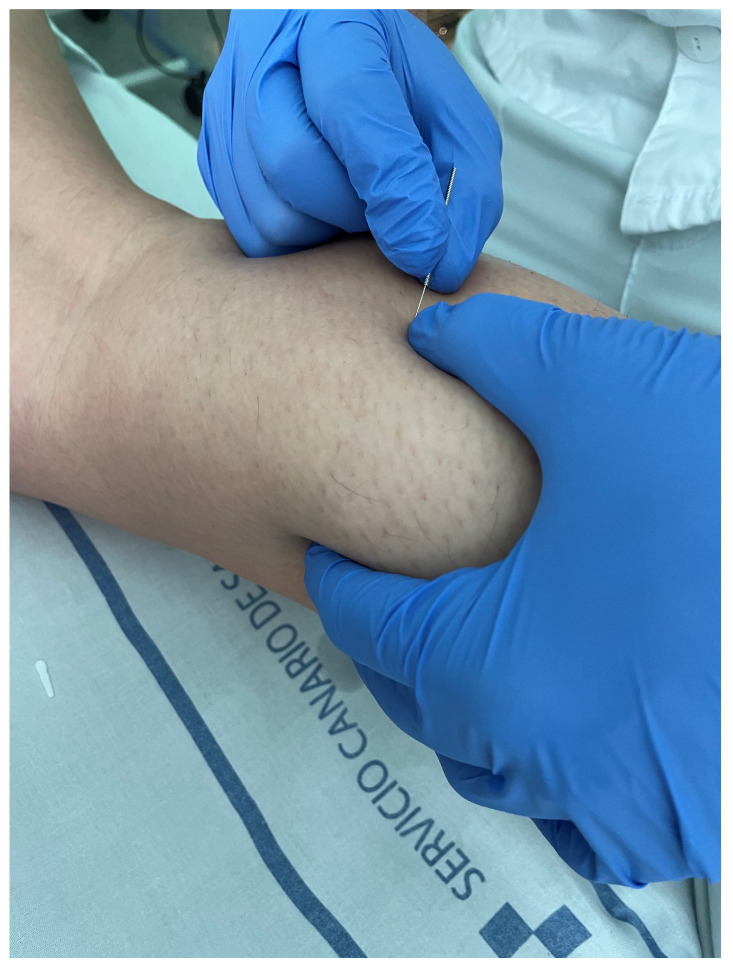
Dry needling on gastrocnemius medialis muscle.

The real DN group received a single session of DN applied to myofascial trigger points in the spastic muscles of the most affected lower limb. Sterile stainless steel filiform needles (0.3 mm × 40 mm, Agupunt^®^, Barcelona, Spain) were inserted using Hong’s fast-in-and-out technique, 1 Hz for approximately 30 s only in gastrocnemius medialis muscle (Figure 1). This muscle was selected due to its frequent involvement in lower-limb spasticity in MS and its well-established contribution to gait impairment.

The sham group received an identical-appearing intervention using a monofilament, without introducing it through the skin but simulating the sensation of the needle for 30 s. Application sites and procedure duration were identical to the active group. All interventions were performed by the same physiotherapist (A.J.O.) with >5 years of experience in DN, certified by the Spanish Society of Invasive Physical Therapy.

### 2.3. Outcome Measures and Responder Definitions

Assessments were conducted at baseline and 4 weeks post-intervention. The following measures were used to define response:

Timed Up-and-Go (TUG): Time (s) to rise from a chair, walk 3 m, turn, return, and sit down. Responder criterion: improvement ≥ 2.0 s, representing approximately 0.5–0.7 standard deviations of change and considered clinically perceptible in MS populations [26].

MSQOL-54 Physical Health Composite Score: Transformed to a 0–100 scale. Responder criterion: improvement ≥ 5 points, corresponding to the standard error of measurement and a clinically detectable change in MS [27].

The 25-Foot Walk Test (T25FW): Time (seconds) to walk 7.62 m at fastest safe speed. Responder criterion: improvement ≥ 10% in time (equivalent to ≥10% increase in gait speed), a sensitive threshold for pilot studies [24].

Additional baseline measures included: EDSS total score and pyramidal subscore (0–5, higher scores indicating greater spasticity) [28]; 12-item Multiple Sclerosis Walking Scale (MSWS-12) [29]; MSQOL-54 Mental Health Composite Score [27]; and demographic/clinical variables (age, sex, body mass index, disease duration, MS type, assistive device use).

The definition of a global responder as meeting at least one outcome-specific threshold was intentionally designed to prioritize sensitivity over specificity. This approach was selected to maximize detection of early response signals in an exploratory pilot context. However, this strategy may increase the risk of false-positive classification, particularly in small samples, and should therefore be interpreted with caution.

### 2.4. Antispastic Medication

All participants were on stable antispastic pharmacological treatment at baseline, including fampridine and/or baclofen. As per the study protocol and inclusion criteria, no changes in antispastic medication type or dosage were permitted during the 6 months prior to enrollment, nor were any modifications allowed before, during, or after the intervention, including the entire follow-up period. This strict control of concomitant medication was implemented to minimize potential pharmacological confounding.

### 2.5. Statistical Analysis

Analyses were performed using SPSS v28.0 (IBM Corp., Armonk, NY, USA) and R v4.3.1. A two-sided *p* < 0.05 was considered statistically significant, though in exploratory analyses all p-values are reported for contextual interpretation. Descriptive statistics included mean (standard deviation, SD) for normally distributed continuous variables, median [interquartile range, IQR] for non-normal variables, and frequency (percentage) for categorical variables. Normality was assessed using Shapiro–Wilk tests. For the primary analysis, proportions of global responders between groups were compared using Fisher’s exact test. Absolute risk difference with 95% confidence interval (CI) was calculated using the Wald method. Within the active group, baseline characteristics of responders versus non-responders were compared using Student’s *t*-tests (or Mann–Whitney U tests) for continuous variables and Fisher’s exact tests for categorical variables. Effect sizes were reported as Cohen’s d (small: 0.2, moderate: 0.5, large: 0.8) for continuous variables and Cramer’s φ for categorical variables. Pearson’s correlation coefficient (r) was calculated between baseline EDSS pyramidal subscore and change in MSQOL-54 physical score in the active group. Post hoc power for detecting the observed responder difference (33.3% vs. 0%) was estimated via Monte Carlo simulation (10,000 replicates). This secondary responder analysis was exploratory in nature and not powered to formally detect predictors of response or between-group differences. All analyses were intended to generate hypotheses rather than to establish efficacy.

## 3. Results

### 3.1. Participant Characteristics

**Figure 2:**
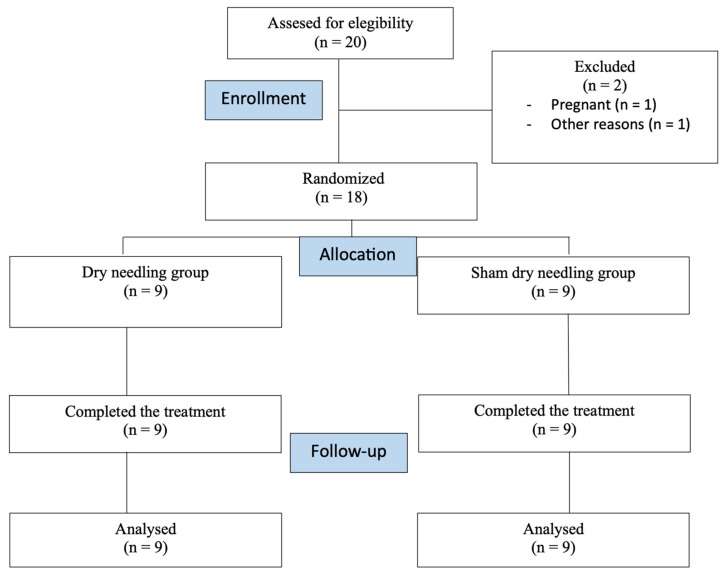
CONSORT 2010 flow diagram of participants throughout the course of the study.

Of 22 patients screened, 18 met eligibility criteria and were randomized (9 per group) (Figure 2). All completed the 4-week assessment (100% retention). Baseline characteristics were comparable between groups (all *p* > 0.05, Table 1). The overall sample (*n =* 18) had a mean age of 47.2 ± 6.8 years, was predominantly female (66.7%), and had median disease duration of 11 [IQR 6–12] years. Most participants (66.7%) had relapsing–remitting MS. Median EDSS was 4.5 ± 1, with mean pyramidal subscore of 3.1 ± 1.0, indicating moderate-to-severe spasticity.

### 3.2. Responder Rates

Applying the sensitive responder criteria, 3 of 9 patients (33.3%, 95% CI: 7.5–70.1%) in the DN group were classified as global responders, compared with 0 of 9 (0%, 95% CI: 0.0–33.6%) in the sham group (*p* = 0.214, Fisher’s exact test) (Table 2). The absolute risk difference was 33.3% (95% CI: −8.6% to 75.2%). At the individual domain level, two patients (22.2%) in the DN group met the MSQOL-54 physical criterion, one (11.1%) met the T25FW criterion, and none met the TUG criterion. In the sham group, two patients (22.2%) met the T25FW criterion but showed worsening in other domains; none met the MSQOL-54 or TUG criteria.

### 3.3. Characteristics of Responders in the Active Group

Within the DN group, responders (*n* = 3) showed patterns suggesting compared to non-responders (*n* = 6) (Table 3). All responders (100%) had relapsing–remitting MS versus 40% of non-responders (*p* = 0.090, Cramer’s φ = 0.58, large effect). Responders had lower baseline EDSS (mean 3.4 vs. 4.4, *p* = 0.222, Cohen’s d = −0.86) and higher pyramidal subscores (3.0 vs. 2.6, *p* = 0.553, Cohen’s d = 0.38). No significant differences were observed in age, sex, disease duration, or baseline functional measures.

### 3.4. Correlation Between Baseline Spasticity and Quality-of-Life Improvement

**Figure 3:**
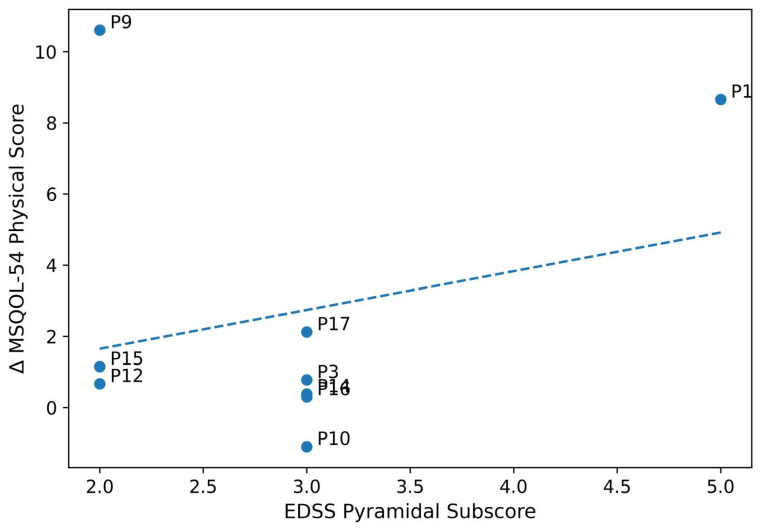
Scatter plot showing correlation between baseline spasticity (EDSS pyramidal subscore) and improvement in physical quality of life (MSQOL-54 physical score change) in the dry needling group. Each point represents one patient (*n* = 9). The solid line indicates the linear regression fit (y = 2.45x − 4.32); shaded area represents 95% confidence interval. Pearson’s r = 0.52, *p* = 0.150. Interpretation of this association is limited by the small sample size and the exploratory nature of the analysis.

In the DN group, a moderate positive correlation was observed between baseline EDSS pyramidal subscore (spasticity severity) and improvement in MSQOL-54 physical score at 4 weeks (r = 0.52, 95% CI: −0.16 to 0.87, *p* = 0.150, R^2^ = 0.27) (Figure 3). This relationship was not present in the sham group (r = −0.12, *p* = 0.760). However, this finding did not reach statistical significance and should be interpreted cautiously given the small sample size.

### 3.5. Sensitivity and Power Analyses

Sensitivity analyses confirmed the robustness of findings. No patient in either group met ≥2 criteria, highlighting the conservative nature of stricter thresholds. Post hoc power analysis indicated that with the observed responder difference (33.3% vs. 0%) and sample size (*n* = 9 per group), the achieved statistical power was 35% for α = 0.05 two-sided. To detect this difference with 80% power, 54 patients per group (108 total) would be required.

## 4. Discussion

This secondary responder-based analysis of a pilot randomized controlled trial provides preliminary evidence that a clinically meaningful proportion of ambulatory people with MS and lower-limb spasticity may benefit from a single session of DN. Approximately one-third of participants in the DN group met predefined responder criteria, compared with no responders in the sham group. Although the between-group difference did not reach conventional statistical significance (*p* = 0.214), the magnitude and direction of the observed effect (33.3% vs. 0%) are clinically relevant and should be interpreted in the context of the exploratory nature, limited sample size, and primary feasibility objectives of the parent trial. Importantly, responder analyses are specifically designed to detect clinically meaningful individual improvements that may not be captured by group-level mean comparisons, particularly in heterogeneous conditions such as MS [30,31].

While responder rates of similar magnitude have been reported for other spasticity interventions, such as botulinum toxin, these figures should not be interpreted as indicating comparable effect sizes. The present study involved a minimal intervention dose (single muscle, single session) and a very small sample, precluding any direct comparison [32,33].

Similarly, rehabilitation-based interventions often demonstrate modest average effects but meaningful benefits in specific subgroups of patients [9]. This heterogeneity likely reflects the multifactorial pathophysiology of spasticity in MS, which involves variable contributions from neural hyperexcitability, altered reflex modulation, muscle stiffness, and biomechanical adaptations, as well as differences in disease phenotype, disability level, and neuroplastic capacity [34,35]. The responder criteria employed in the present study—≥2.0 s improvement in the Timed Up and Go (TUG), ≥10% improvement in the Timed 25-Foot Walk (T25FW), and ≥5-point improvement in the Multiple Sclerosis Quality of Life-54 (MSQOL-54)—were selected to balance sensitivity for signal detection with established thresholds of clinical meaningfulness. These cut-offs are consistent with prior work defining minimal clinically important differences in mobility and patient-reported outcomes in MS and other neurological populations [24,36,37].

Nevertheless, it is possible that the TUG threshold of ≥2.0 s was relatively stringent for detecting short-term changes following a single-session intervention. Future studies may benefit from incorporating additional outcomes with higher responsiveness to acute neuromuscular modulation, such as instrumented gait parameters, spatiotemporal variability, or patient-reported measures of perceived stiffness and ease of movement [38,39]. The finding that responders were more likely to have a relapsing–remitting disease course and lower baseline disability is biologically plausible and aligns with the existing literature. Individuals with relapsing–remitting MS generally exhibit greater preservation of axonal integrity, higher functional reserve, and enhanced capacity for activity-dependent neuroplasticity compared with those with progressive phenotypes [3,22,40]. These factors may facilitate more immediate functional gains following interventions that modulate neuromuscular excitability, such as DN. Although the present study was not powered to formally test predictors of response, the observed pattern supports the hypothesis that disease phenotype and baseline disability may influence responsiveness to DN and warrants prospective investigation in adequately powered trials.

Additionally, the moderate positive correlation observed between baseline spasticity severity and improvement in quality of life suggests that individuals with greater initial impairment may experience larger perceived benefits. This finding is consistent with prior rehabilitation research demonstrating that patients with greater “room for improvement” often show larger gains in patient-reported outcomes, even when objective functional changes are modest [41,42]. The observed responder patterns must be interpreted with caution. Given the small sample size, wide confidence intervals, and exploratory nature of the analysis, the possibility of false-positive findings or chance-driven patterns cannot be excluded. Responder-based approaches are sensitive to threshold selection and post hoc interpretation, particularly in heterogeneous conditions such as MS.

Importantly, quality of life is a multidimensional construct influenced not only by physical function but also by symptom burden, fatigue, perceived control, and participation, all of which may be indirectly affected by changes in spasticity [43]. From a clinical perspective, these findings support the potential role of DN as a personalized adjunctive intervention for a subset of people with MS rather than as a universal treatment. The preliminary identification of response-associated characteristics may help clinicians tailor interventions more effectively and avoid unnecessary treatments in individuals unlikely to benefit. One possible implementation strategy is a “test–retest” or trial-response approach, whereby a single DN session is used to assess short-term responsiveness before initiating a longer treatment program. Such an approach has been proposed for other focal spasticity interventions and aligns with principles of precision rehabilitation [44].

Several limitations should be acknowledged. First, the small sample size limits statistical power and precludes definitive conclusions regarding efficacy or predictors of response. Second, the responder thresholds, while grounded in the literature, have not been specifically validated for DN interventions in MS and should be interpreted cautiously. Third, the use of a single treatment session likely underestimates the potential cumulative effects of repeated DN applications, which may produce more robust and sustained changes in neuromuscular function [16]. Another important limitation is the absence of direct objective measures of spasticity, such as the Modified Ashworth Scale, electromyographic assessments, or reflex-based measures. Although the EDSS pyramidal subscore provides a clinically meaningful estimate of spasticity-related impairment in multiple sclerosis, it does not allow detailed mechanistic interpretation of neuromuscular changes. The use of a single muscle and single session was intentional, aligning with a ‘test-dose’ approach to evaluate short-term responsiveness before considering repeated or multi-muscle protocols. Consequently, the generalizability of the findings to standard clinical settings is limited. Despite these limitations, this study has several notable strengths. The randomized, double-blind, sham-controlled design minimizes bias and strengthens internal validity. Participant retention was 100%, supporting feasibility and acceptability. Validated functional and quality-of-life outcomes were used, and the responder-based analytical framework was pre-specified, reducing the risk of post hoc interpretation. Furthermore, the observed association between baseline spasticity severity and outcome improvement provides preliminary mechanistic support for the intervention and generates testable hypotheses for future research. Taken together, these findings should not be interpreted as evidence of efficacy, but rather as preliminary signals identifying potential response heterogeneity and informing the design of future confirmatory trials.

## 5. Conclusions

This secondary responder analysis suggests that, in this exploratory sample, approximately one-third of patients with MS and spasticity may experience clinically relevant benefits from a single session of DN, with relapsing–remitting phenotype and lower baseline disability as potential predictors. The moderate correlation between baseline spasticity and quality-of-life improvement supports the biological plausibility of the intervention. While these findings are preliminary due to sample size limitations, they provide valuable signals for personalizing spasticity management in MS. These findings should not be interpreted as evidence of efficacy, but rather as preliminary signals highlighting response heterogeneity and informing future study design. Future adequately powered RCTs should stratify by MS type and spasticity severity to confirm these predictive profiles and optimize patient selection for DN therapy.

## Figures and Tables

**Table 1:** Baseline characteristics of randomized participants. Abbreviations: BMI, body mass index; RRMS, relapsing–remitting multiple sclerosis; EDSS, Expanded Disability Status Scale; T25FW, 25-Foot Walk Test; TUG, Timed Up-and-Go; MSQOL-54, Multiple Sclerosis Quality of Life-54; MSWS-12, 12-item Multiple Sclerosis Walking Scale; SD, standard deviation; IQR, interquartile range.

Characteristic	Total (*n* = 18)	Dry Needling (*n* = 9)	Sham Needling (*n* = 9)	*p*-Value
Age (years) Mean ± SD	47.2 ± 6.8	46.4 ± 9.8	48.0 ± 2.8	0.651
Female (%)	12 (66.7)	6 (66.7)	6 (66.7)	1
BMI (kg/m^2^) Mean ± SD	24.9 ± 3.9	23.1 ± 3.4	25.5 ± 4.3	0.310
Disease duration (years) Median	11	12	11	0.847
RRMS (5)	12 (66.7)	6 (66.7)	6 (66.7)	1
EDSS total Median [IQR]	4.5 [1.0]	4.94 [1.21]	3.83 [1.2]	0.400
EDSS pyramidal subscore Median [IQR]	3.1 [1.0]	2.9 [0.9]	3.2 [1.1]	0.521
T25FW (s) Mean ± SD	8.1 ± 3.3	7.1 ± 2.1	9.1 ± 4.1	0.225
TUG (s) Mean ± SD	11.1 ± 4.2	9.9 ± 2.5	12.1 ± 5.2	0.272
MSQOL-54 physical Mean ± SD	53.4 ± 10.4	51.8 ± 22.8	55.0 ± 18.8	0.758
MSQOL-54 Mental Mean ± SD	57.3 ± 19.5	60.7 ± 18.4	54.0 ± 20.8	0.488
MSWS-12 Mean ± SD	35.4 ± 11.8	31.3 ± 12.8	39.4 ± 9.2	0.140

**Table 2:** Responder analysis at 4 weeks post-intervention. Fisher’s exact test. CI, confidence interval. * *p*-Value < 0.05.

Outcome/Criterion	Dry Needling (*n* = 9)	Sham Needling (*n* = 9)	Absolute Difference (95% CI)	*p*-Value *
By Domain				
T25FW (≥10% improvement)	1 (11.1%)	2 (22.2%)	−11.1% (−46.7 to 24.4%)	1
TUG (≥2.0 s improvement)	0	0	0	-
MSQOL-54 Physical (≥5-point improvement)	2 (22.2%)	0	22.2% (−9.1 to 53.5%)	0.476
Global Responder (≥1 criterion)	3 (33.3%)	0	33.3% (−8.6% to 75.2%)	0.214

**Table 3:** Baseline characteristics of responders versus non-responders in the dry needling group. Abbreviations: CI, confidence interval; SD, standard deviation; RRMS, relapsing–remitting multiple sclerosis; EDSS, Expanded Disability Status Scale; T25FW, 25-Foot Walk Test; TUG, Timed Up-and-Go; d, Cohen’s d; φ, Cramer’s φ.

Characteristic	Responders (*n* = 3)	Non-Responders (*n* = 6)	Difference (95% CI)	*p*-Value	Effect Size
Age (years) Mean ± SD	47.2 ± 9.1	46.4 ± 9.8	1.1 (−13.2 to 15.4)	0.874	d = 0.11
Female (%)	2 (66.7)	3 (50)	16.7% (−55.1 to 88.5%)	1	φ = 0.14
Disease duration Median	9.5	10.8	−1.3	0.792	d = −0.17
RRMS (5)	3 (100)	2 (40)	60% (10.1% to 100%)	0.090	φ = 0.58
EDSS total Mean ± SD	3.4 ± 0.9	4.0 ± 1.3	−1 (−2.8 to 0.8)	0.222	d = −0.86
EDSS pyramidal subscore Mean ± SD	3.0 ± 1.2	2.6 ± 0.9	0.4 (−1.2 to 2.0)	0.553	d = 0.38
T25FW (s) Mean ± SD	7.1 ± 2.3	7.1 ± 2.1	0.0 (−2.7 to 2.7)	0.953	d = 0.00
TUG (s) Mean ± SD	10.2 ± 2.5	9.8 ± 2.7	0.4 ± (−2.9 to 3.7)	0.819	d = 0.15
MSQOL-54 physical Mean ± SD	53.4 ± 23.8	51.1 ± 24.4	2.3 (−30.1 to 34.7)	0.888	d = 0.10

## Data Availability

The data presented in this study are available on request from the corresponding author due to ethical restrictions and the protection of participants’ privacy.
